# Optimal treatment selection for hepatocellular carcinoma in the era of immunotherapy

**DOI:** 10.1007/s00535-026-02411-7

**Published:** 2026-04-20

**Authors:** Chen-Ta Chi, Yi-Hsiang Huang

**Affiliations:** 1 Division of Gastroenterology and Hepatology, Department of Medicine, Taipei Veterans General Hospital, Taipei, Taiwan; 2 Institute of Clinical Medicine, School of Medicine, National Yang Ming Chiao Tung University, Taipei, Taiwan; 3 Department of Medical Research, Taipei Veterans General Hospital, 201 Shih-Pai Road, Sec. 2, 112201 Taipei, Taiwan

**Keywords:** HCC, Immunotherapy, Optimal treatment, Biomarker, AI

## Abstract

Immunotherapy has revolutionized hepatocellular carcinoma (HCC) management, necessitating personalized strategies in current guidelines. Despite curative intent for early-stage HCC, recurrence rates remain high. While antiviral therapy mitigates risk, no adjuvant therapy is currently established for high-risk patients. Artificial intelligence (AI) models now offer promising tools for predicting post-resection recurrence.

For intermediate-stage HCC, the 7–11 criteria and radiological patterns guide treatment selection. Combining transarterial chemoembolization (TACE) with tyrosine kinase inhibitors has already improved objective response rates (ORR) and progression-free survival (PFS) over TACE monotherapy. Furthermore, the Phase III EMERALD-1 and LEAP-012 trials demonstrated that TACE plus immunotherapy significantly extends PFS. The TALENTACE trial further investigates this synergy, evaluating the efficacy and safety of TACE combined with immune checkpoint inhibitors (ICIs) combination therapy to optimize outcomes in this population.

Achieving a cancer-free state through curative conversion—utilizing liver-directed, systemic, or combination therapies—remains a primary clinical goal. While ICIs are the standard for advanced HCC, reliable biomarkers are still lacking. Emerging evidence suggests that the tumor microenvironment, genetic signatures, and the gut microbiota-metabolite axis are significantly associated with ICI outcomes. Integrating these complex biological features through machine learning holds the potential to identify robust biomarkers, ultimately refining immunotherapy selection and patient prognosis.

## Introduction

Primary liver cancer is the sixth most common cancer worldwide and the third leading cause of cancer-related death globally [1]. Hepatocellular carcinoma (HCC) is the most common type of primary liver cancer, accounting for approximately 80% of liver cancer cases [2]. The incidence of HCC varies significantly between genders and races, with the highest incidence in East Asia [1, 3, 4]. Chronic hepatitis B virus (HBV) and hepatitis C virus (HCV) remain the major etiological risk factors, but antiviral treatment of HBV and HCV can significantly reduce the risk of HCC [1]. Meanwhile, the incidence and mortality of HCC related to alcoholic and metabolic dysfunction-associated steatotic liver disease (MASLD) have increased, and MASLD has become the leading cause of HCC in the absence of cirrhosis [1, 5–8]. Furthermore, the primary risk factor for HCC is cirrhosis, regardless of the underlying etiology. In patients with cirrhosis, the annual incidence of HCC is approximately 2% [9].

Immunotherapy has revolutionized the treatment of HCC, particularly for advanced-stage patients, by leveraging the immune system to target cancer cells. The role of immunotherapy at each stage has also been addressed in various clinical trials and treatment guidelines [1, 10–13]. Nevertheless, several challenges remain: optimizing the combination of immune checkpoint inhibitors (ICIs) with liver-directed therapies to overcome resistance, identifying predictive biomarkers of response, and expanding the clinical utility of ICIs into the neoadjuvant and adjuvant settings for early-stage HCC.

In this review, we examine the current landscape of immunotherapy across the clinical stages of HCC: the emerging role and inherent challenges of adjuvant therapies in early-stage disease; strategies for integrating ICIs with other modalities to enhance efficacy in intermediate-stage HCC; and the search for predictive biomarkers and TME-modulating strategies to guide treatment in advanced-stage cases.

## Immunotherapy in early-stage HCC: current challenges

Management of very early-stage HCC (BCLC 0) primarily utilizes RFA or MWA, while surgical resection is reserved for specific clinical indications. In BCLC A patients with tumors ≤ 2 cm, RFA and resection yield equivalent long-term survival. Conversely, for tumors larger than 2 cm, the increased risk of local recurrence and incomplete response with ablation makes surgical resection the superior therapeutic approach [1, 11, 12].

### High recurrence rate after surgical resection or RFA of early-stage HCC

The natural history of non-resectable HCC reveals poor outcomes, with a 3-year survival rate of only 28% [14]. For patients with Child–Pugh A liver function and early-stage HCC, hepatic resection offers favorable long-term survival; however, the persistent risk of late recurrence remains a major clinical challenge [15]. Similarly, while RFA serves as an effective first-line alternative for non-surgical candidates, its 5-year cumulative incidence of new tumor emergence reaches up to 81% [16]. Despite this, RFA remains superior to percutaneous ethanol injection (PEI) in improving overall survival and delaying tumor progression [17]. Postoperative recurrence typically follows a bimodal pattern [18]. Early recurrences, peaking within the first year, are generally attributed to intrahepatic micrometastases from the primary tumor. In contrast, late recurrences (4 to 5 years post-treatment) typically arise from multicentric carcinogenesis, driven by the underlying “field effect” of the cirrhotic liver.

### Antiviral therapy and HCC recurrence

Hepatitis B virus (HBV) DNA levels represent critical determinants for both primary hepatocarcinogenesis and post-resection recurrence [19]. Although large-scale cohort studies demonstrate that early initiation of antiviral therapy significantly reduces overall mortality, such therapy may not independently improve long-term (10-year) recurrence-free survival (RFS) [20]. This suggests that while viral suppression mitigates liver-related death, it may not fully counteract the intrinsic risk of recurrence. Beyond virological factors, host immune status profoundly influences oncological outcomes. Specifically, lower baseline serum interferon-gamma (IFN-γ) levels correlate with a higher recurrence risk, highlighting its potential as a prognostic biomarker following curative treatments [21].

### Adjuvant anti-cancer therapy

Adjuvant therapy aims to activate T cells post-surgery, whereas neoadjuvant therapy primes a diverse immune response prior to resection [22, 23]. Currently, there is no established adjuvant therapy, especially for patients at high risk of HCC recurrence after radical resection or ablation. In the era of tyrosine kinase inhibitors (TKIs), adjuvant TKI therapy has emerged as a potential strategy to mitigate the risk of recurrence following curative resection or ablation. The STORM trial—a phase 3, randomized, double-blind, placebo-controlled study—evaluated sorafenib (SORA) versus placebo as an adjuvant treatment for patients with HCC HCV after surgical resection or local ablation. The trial demonstrated no significant difference in median recurrence-free survival (RFS) between the two cohorts, reported at 33.3 months for the sorafenib group compared to 33.7 months for the placebo group (hazard ratio [HR] = 0.940; *p* = 0.26) [24]. The STORM trial’s failure is attributed to its “all-comers” design, lacking enrichment for high-risk patients. Consequently, subsequent phase 3 clinical trials have pivoted their designs to exclusively target high-risk populations following curative resection or ablation, aiming to optimize the therapeutic index of adjuvant interventions.

The IMbrave050 study, a randomized, open-label, multicenter, phase 3 trial, assesses the efficacy of adjuvant atezolizumab plus bevacizumab (Atezo/Bev) versus active surveillance in patients with resected or ablated high-risk HCC. At the pre-specified interim analysis, adjuvant Atezo/Bev significantly improved RFS compared with active surveillance ([HR] = 0.72; *p* = 0.012) with a median follow-up of 17.4 months [25]. However, in the final updated analysis, with a median follow-up of 35.1 months, the initial RFS benefit of Atezo/Bev compared with active surveillance was not sustained ([HR] = 0.90), and the benefit-risk profile did not support Atezo/Bev as adjuvant therapy for all high-risk HCCs. Nevertheless, RFS among resection patients was numerically better in who were beyond the up-to-7 criteria [26]. More recently, data from the KEYNOTE-937 trial—presented at the 2026 ASCO GI Annual Meeting—demonstrated that pembrolizumab monotherapy failed to meet its primary endpoint of recurrence-free survival (RFS) for the high-risk group [27]. The trial reported a median RFS of 46.7 months for the pembrolizumab group compared to 45.5 months for the placebo group (HR = 1.06; *p* = 0.719).

Although IMbrave050 and KEYNOTE-937 both targeted patients at high risk of HCC recurrence, their inclusion criteria were not identical. In general, KEYNOTE-937 adopted a broader definition of risk, incorporating a larger proportion of the intermediate-risk group (such as patients with solitary tumors 2–5 cm without microvascular invasion) compared to the more stringent high-risk requirements of IMbrave050 (Table 1). The favorable 1-year RFS observed in IMbrave050, particularly the superior outcomes among patients with high tumor burden, suggests that adjuvant immunotherapy retains significant potential for the “true” high-risk population. These findings indicate that while monotherapy may falter, intensified combination regimens with bevacizumab may be necessary to effectively address the high recurrence rates associated with advanced clinicopathological features.

**Table 1:** Data of adjuvant, neoadjuvant, and perioperative immunotherapy for HCC: patient selection criteria and outcomes

Treatment Setting	Trial (Phase)	Treatment arm (*n*)	Target population	Median RFS, mo	Hazard ratio (95% CI)	Median OS, mo	Hazard ratio (95% CI)
**Adjuvant**	KEYNOTE-937 (Phase 3)	Pembro (476)	*Intermediate to high risk of recurrence* 1. *Resection* Single tumor, 2-5 cm, no VI Single tumor > 5 cm; Single tumor with VI or satellite nodules; 2–3 tumors, each ≤ 3 cm; Poorly differentiated tumor (any size) Single tumor with VP1/2; ≥ 4 tumors, each ≤ 5 cm; 2–3 tumors, at least one > 3 cm but ≤ 5 cm 2. *Ablation* Single tumor > 2 cm but ≤ 5 cm 2–3 tumors, each ≤ 3 cm	46.7	1.06 (0.88–1.26)	NR	1.08 (0.81–1.43)
		Placebo (483)		45.5	–	NR	–
	IMbrave050 (Phase 3)	Atezo/Bev (334)	*High-risk of recurrence*: 1. *Resection*: ≤ 3 tumors, the largest > 5 cm, or poor differentiation (grade 3 or 4) ≥ 4 tumors, all ≤ 5 cm ≤ 3 tumors, the largest ≤ 5 cm with mVI or Vp1/Vp2 2. *Ablation*: Single tumor > 2 cm but ≤ 5 cm Up to 4 tumors, all ≤ 5 cm	33.2	0.90 (0.72–1.22)	NR	1.26 (0.85–1.87)
		None (334)		36.0	–	NR	–
**Neoadjuvant**	Ho et al. (Phase 1b)	Cabo/Nivo (15)	Borderline resectable/Locally advanced: single tumor > 5 cm; unilobar multifocal disease (> 3 tumors or one tumor > 3 cm); bilobar disease; high-risk features (tumor > 3 cm with MVI)	NR^*^ (DFS)	(Single-arm)	NR	(Single-arm)
	Lin et al. (Phase 2)	Nivo/Ipi (43)	Potential resectable (≥ 1 criteria): MVI; > 3 tumors; multiple tumors (< 5 cm) with bilateral involvement or significant portal hypertension; high risk of recurrence after surgery (e.g. direct diaphragmatic invasion)	Resection rate: 55.8% Risk of recurrence: 33%	(Single-arm)	NR	(Single-arm)
**Perioperative**	CARES-009 (Phase 2/3)	Camre/Rivo (148)	Resectable (Intermediate or High-risk)^#^: Intermediate risk: CNLC stage Ib–IIa High risk: CNLC stage IIb–IIIa	42.1 (EFS)	0.59 (0.41–0.85)	NR	Immature
		Surgery alone (146)		19.4 (EFS)	–	NR	–

Hazard ratios (HR) are presented for the experimental group versus the control group

Abbreviations: *Atezo/Bev* atezolizumab plus bevacizumab, *BCLC* Barcelona Clinic Liver Cancer, *Cabo/Nivo* cabozantinib plus nivolumab, *Camre/Rivo* camrelizumab plus rivoceranib, *CI* confidence interval, *CNLC* China Liver Cancer Staging, *DFS* disease-free survival, *EFS* event-free survival, *HCC* hepatocellular carcinoma, *IVC* inferior vena cava, *mo* months, *MVI* macrovascular invasion, *mVI* microvascular invasion, *NA* not applicable, *Nivo/Ipi* nivolumab plus ipilimumab, *NR* not reached/not reported, *OS* overall survival, *Pembro* pembrolizumab, *PFS* progression-free survival, *RFS* recurrence-free survival, *Vp* portal vein invasion

*12 of 15 enrolled patients successfully underwent surgical resection and were included in the DFS analysis; the median DFS was not reached

#This corresponds to BCLC A [single tumor > 5 cm], BCLC B/C without Vp4 involvement or extrahepatic metastasis

### Neoadjuvant and perioperative immunotherapy

Neoadjuvant immunotherapy has emerged as a promising strategy, leveraging the presence of the primary tumor to prime a robust, systemic anti-tumor immune response prior to surgical intervention. By initiating treatment while the full repertoire of tumor-associated antigens is present, this approach aims to eradicate micrometastatic disease and reduce the risk of postoperative recurrence. However, clinical data from large-scale phase 3 trials remain immature, and the long-term impact on OS has yet to be fully established.

Early-phase evidence shows potential; for instance, a Phase 1b study by Ho et al. evaluating cabozantinib plus nivolumab in 15 patients with borderline resectable or locally advanced HCC demonstrated encouraging results, with median disease-free survival (DFS) not reached among the 12 patients who underwent successful resection [28]. Furthermore, Lin et al. investigated neoadjuvant nivolumab plus ipilimumab in 43 patients with potentially resectable HCC; among the 24 patients who proceeded to surgery, 8 (33.3%) achieved a major pathological response (MPR, defined as > 90% tumor necrosis). Notably, however, 8 patients experienced tumor recurrence by the data cutoff (Table 1) [29].

The theoretical foundation for perioperative immunotherapy is compelling: initiating treatment while the primary tumor remains intact allows the immune system to recognize a full repertoire of tumor-associated antigens, thereby priming a robust systemic anti-tumor T-cell response prior to surgical intervention. Subsequent postoperative immunotherapy provides a critical ‘immune boost’ that sustains T-cell activation, which is essential for eradicating residual micrometastatic disease and preventing recurrence. This approach was recently validated by the landmark CARES-009 phase 2/3 trial, which demonstrated that perioperative camrelizumab plus rivoceranib significantly improved median event-free survival (EFS) compared to surgery alone (42.1 vs. 19.4 months; HR 0.59) in patients with intermediate-to-high-risk HCC, while also achieving higher major pathological response rates of 35% defined as ≤ 50% viable tumor cells in resected specimens (Table 1) [30].

In general, neoadjuvant immunotherapy potentially allows for parenchymal-sparing resections, which are vital for HCC patients with underlying cirrhosis. However, there are several unresolved issues, including how many cycles are optimal, the lack of a definitive “liquid biopsy” to confirm molecular clearance post-neoadjuvant therapy, as well as the optimal threshold of major pathological response (< 10% or < 50% viable tumor cells) that can correlate with EFS and overall survival.

### Radiomics and machine-learning model to select a high-risk group

The success of adjuvant, neoadjuvant, and perioperative immunotherapy clinical trials relies heavily on the precise selection of patients at high risk for recurrence. However, current prediction models largely dependent on traditional clinicopathological features remain suboptimal for identifying early recurrence after surgical resection. This diagnostic gap underscores the need for more robust biomarkers and integrated scoring systems to better stratify patients for intensified perioperative interventions.

While radiomic signatures have been investigated as independent prognostic biomarkers in various malignancies, their application in HCC is rapidly evolving [31]. Recently, Lee et al. proposed a novel evolutionary learning-derived method called Genetic Algorithm for predicting Recurrence after Surgery of Liver cancer (GARSL) with interpretability using both clinical and radiomic features to predict early recurrence of HCC after surgical resection [32]. A total of 143 features, including 26 preoperative clinical features, 5 postoperative pathological features, and 112 radiomic features, were used to develop GARSL preoperative and postoperative models. The areas under the receiver operating characteristic curve (AUROC) for early recurrence of HCC within 2 years were 0.781 and 0.767 in the training set, and 0.739 and 0.741 in the test set, respectively. The GARSL model—an evolutionary learning-derived clinical-radiomic framework—has demonstrated significantly higher accuracy in predicting early HCC recurrence compared to the traditional ERASL models (*p* < 0.001) [32]. This finding suggests that future clinical trials in the adjuvant and neoadjuvant settings may increasingly rely on artificial intelligence (AI)-based selection criteria to precisely identify the high-risk patient populations most likely to benefit from systemic intervention.

## Intermediate-stage HCC: how to improve treatment outcome?

BCLC Stage B is characterized by multifocal HCC in patients with preserved liver function (Child–Pugh A/B), an ECOG performance status of 0, and the absence of vascular invasion or extrahepatic spread. Despite transarterial chemoembolization (TACE) being the historical mainstay, tumor burden at this stage is highly heterogeneous. Consequently, the updated BCLC staging system now stratifies patients based on specific tumor burden and morphology to provide tailored treatment recommendations. Notably, these updates emphasize the role of downstaging strategies—utilizing both intra-arterial therapies and systemic agents—to convert patients toward curative-intent treatments such as resection or liver transplantation [1, 11–13, 33].

### TACE refractoriness and unsuitability

The 2014 updated Japan Society of Hepatology (JSH) Consensus firstly defines TACE failure/ refractoriness as follows [34]: (1) Two or more consecutive TACE treatments were ineffective, even if the chemotherapeutic agents were changed and/or the feeding artery is reanalyzed, or the appearance of more lesions in the liver than the number of lesions recorded at the previous TACE procedure; (2) Continuous elevation of tumor markers right after TACE even though transient minor reduction is observed; (3) Appearance of vascular invasion; (4) Appearance of extrahepatic spread. If the patient is refractory to TACE, advanced-stage treatment such as systemic therapy is recommended. The 2020 Asia–Pacific Primary Liver Cancer Expert (APPLE) Consensus statement further defines TACE-unsuitability as each one of the following three clinical conditions [33]: (1) Unlikely to respond to TACE: confluent multinodular type, massive or infiltrative type, simple nodular type with extranodular growth, poorly differentiated type, intrahepatic multiple disseminated nodules, or sarcomatous changes after TACE (2) Likely to develop TACE failure/refractoriness: up-to-7 criteria out nodules (3) Likely to become Child–Pugh B or C after TACE: up-to-7 criteria out nodules (especially, bilobar multifocal HCC) and liver function with mALBI grade 2b.

In general, TACE ORR varies widely (21–85%), with often unsatisfactory OS [33, 35]. A systematic review included 101 studies involving 10,108 patients with HCC who underwent TACE with lipiodol. The median OS was 19.4 months, meaning that approximately one-third of patients had a poor prognosis after 1 year [35]. Therefore, most patients require repeated TACE. However, the ORR decreases with subsequent cycles, and the risk of morbidity and mortality associated with TACE-induced risks increases [33].

For intermediate-stage HCC, the definition of high tumor burden remains controversial. Hung et al. proposed a new 7–11 criteria that combines the number of tumors and the size of the largest tumor, which defines high tumor burden as the sum > 11, intermediate tumor burden as the sum > 7 but no more than 11, and low tumor burden as the sum no more than 7. The new 7–11 criteria had the best discriminative power in predicting radiographic response and survival compared with other traditional definitions of tumor burden [36]. On the other hand, radiographic patterns also determine whether TACE is unsuitable. Hung et al. further investigated the influence of radiologic morphology on the outcomes of initial and subsequent TACE. For both initial and subsequent TACE, encapsulated nodular type HCC had the highest ORR, whereas infiltrative type HCC had the lowest ORR. In the multivariate analysis, radiologic features were significant independent predictors of ORR and OS after receiving subsequent TACE. Therefore, systemic therapy should be considered for patients with intermediate-stage HCC with unfavorable radiologic patterns [37]. The concepts of high tumor burden and unfavorable radiographic patterns have been integrated into current clinical guidelines to refine the management of intermediate-stage HCC (BCLC B). For patients presenting with these features—where the efficacy of TACE is limited—systemic therapy is now recommended as the primary intervention over TACE [1, 11–13, 38].

### TACE combined with TKI for intermediate-stage HCC

At first, the TACTICS trial, a randomized, multicenter prospective trial, compared the efficacy and safety of TACE plus SORA with TACE alone. Patients in the combination group received SORA 400 mg once daily for 2–3 weeks before TACE, followed by 800 mg once daily during on-demand conventional TACE sessions until the time to untreatable (unTACEable) progression (TTUP), defined as untreatable tumor progression, transient deterioration to Child–Pugh C, or appearance of vascular invasion/extrahepatic spread. Although the interim analysis showed that the median progression-free survival (PFS) was significantly longer in the TACE plus SORA group than in the TACE alone group (25.2 months vs 13.5 months; *p* = 0.006) [39], the co-primary design was not met in the final analysis [40]. The updated OS was 36.2 months with TACE plus SORA and 30.8 months with TACE alone ([HR] = 0.861; *p* = 0.40), while the updated PFS was 22.8 months with TACE plus SORA and 13.5 months with TACE alone ([HR] = 0.661; *p* = 0.02) [40]. Notably, post hoc analysis showed that HCC patients with tumor burden beyond up-to-7 criteria had both PFS and OS benefits.

Subsequently, a phase 2, prospective, multicenter, single-arm trial, the TACTICS-L trial, investigated the efficacy and safety of TACE plus lenvatinib (LEN), a drug that more strongly promotes vascular normalization and has a higher ORR than SORA [41]. LEN was to be administered 14–21 days before the first TACE, was stopped 2 days before TACE, and was resumed 3 days after TACE. Repeated TACE is allowed on demand. The primary endpoint of median PFS was 28 months after a minimum of 24 months of follow-up. The secondary endpoint of median OS was not reached [41]. Four weeks after the first TACE, a significant proportion of patients responded to LEN-TACE (ORR, 79.0%) even in patients with poor radiologic patterns, and over half of the patients achieved complete tumor response (CR, 53.2%). Finally, LEN-TACE achieved a high ORR and CR rate (best response: ORR 88.7%, CR rate 67.7% by Response Evaluation Criteria in Cancer of the Liver (RECICL); best response: ORR 85.5%, CR rate 66.1% by Modified Response Evaluation Criteria in Solid Tumors (mRECIST)) [41]. The efficacy outcomes of these key trials evaluating TACE combined with tyrosine kinase inhibitors (TKIs) are summarized in Table 2.

**Table 2:** Comparison of clinical trials evaluating the efficacy of TACE combined with TKIs in intermediate-stage HCC

Trial	Regimen	ORR^+^, %	mPFS^#^, months (HR)	mOS, months (HR)
**TACTICS**	Sorafenib + on-demand TACE	71.3% (RECICL)	22.8 (0.661)	36.2 (0.861)
	TACE alone	61.8% (RECICL)	13.5	30.8
**TACTICS-L**	Lenvatinib + on-demand TACE	85.5% (mRECIST)	28	NR
	–	–	–	–

Abbreviations: *HCC* hepatocellular carcinoma, *HR* hazard ratio, *mo* months, *mOS* median overall survival, *mPFS* median progression-free survival, *mRECIST* Modified response evaluation criteria in solid tumors, *NR* not reached, *ORR* objective response rate, *RECICL* Response Evaluation Criteria in Cancer of the Liver, *TACE* transarterial chemoembolization, *TKI* tyrosine kinase inhibitor

+ORR was assessed using RECICL criteria for TACTICS and mRECIST for TACTICS-L.

#mPFS in the TACTICS and TACTICS-L trials was defined as the time to “unTACEable” progression

### TACE combined with immunotherapy for intermediate-stage HCC

Locoregional therapy (LRT) for HCC plays a pivotal role in modulating tumor immunity by fundamentally altering the tumor microenvironment (TME). The induction of tumor cell necrosis via LRT triggers the release of a diverse repertoire of tumor-associated antigens (TAAs) and damage-associated molecular patterns (DAMPs). This “pro-inflammatory cell death” facilitates the recruitment and maturation of dendritic cells, which subsequently prime T-cell responses. Consequently, LRT can effectively reprogram a traditionally immunosuppressive (or “cold”) TME into an immunosupportive (or “hot”) setting, potentially synergistic with systemic immunotherapy [42, 43].

The EMERALD-1 trial is a phase 3, randomized, placebo-controlled study of TACE combined with durvalumab (Durva) with or without concomitant bevacizumab (Bev) in patients with unresectable HCC (uHCC) who were candidates for embolization [44]. Notably, this study enrolled patients with different stages of BCLC, including stage A (25.8%), stage B (57.3%), and stage C (16.1%). The primary endpoint was met for Durva + Bev + TACE (mPFS 15.0 vs. 8.2 months; HR 0.77; *p* = 0.032), though Durva + TACE showed no significant benefit (mPFS 10.0 months; HR 0.94). The ORR was 43.6%, 41.0%, and 29.6% for Durva + Bev + TACE, Durva + TACE, and TACE, respectively. Therefore, Durva + Bev + TACE became the first ICI-based regimen in a global phase 3 trial to show statistically significant and clinically meaningful improvement in PFS for embolization-eligible uHCC [44].

On the other hand, LEAP-012, a randomized, multicenter, double-blind, phase 3 trial, evaluated the efficacy of LEN + pembrolizumab (Pembro) + TACE vs. placebo + TACE in intermediate-stage HCC. At the interim analysis, PFS was significantly improved for LEN + Pembro + TACE vs TACE (median PFS was 14.6 months vs 10.0 months, [HR] = 0.66, *p* = 0.0002). The OS was immature, and the significance threshold was not met ([HR] = 0.80; *p* = 0.0867). The ORR was 46.8% vs. 33.3% for LEN + Pembro + TACE vs. TACE, respectively [45]. Table 3 summarizes the ORR, mPFS, and mOS between the EMERALD-1 trial and the LEAP-012 trial. Overall, TACE combined with immunotherapy can improve ORR by 12–14% and PFS by 4.6–5 months compared with TACE alone. However, the anticipated survival advantage of combining TACE with immunotherapy remains unsupported by current phase 3 OS data. Furthermore, the recent TALENTACE trial demonstrated that the combination of atezolizumab/bevacizumab plus on-demand TACE significantly prolonged PFS compared to TACE alone (10.3 vs. 6.4 months; HR 0.64). This benefit was specifically observed in patients with a high tumor burden (sum of tumor size and number ≥ 6); however, no significant difference in OS has been observed to date [46].

**Table 3:** Comparison of clinical trials evaluating the efficacy of TACE combined with immunotherapy in intermediate-stage HCC

**Trial**	**Group/Regimen**	**ORR, %** (RECIST 1.1)	**mPFS, months (HR)**	**mOS, months (HR)**
**EMERALD-1**	Durva + Bev + TACE	43.6	15.0 (0.77)	NA
	Durva + TACE	41.0	10.0 (0.94)	NA
	TACE alone	29.6	8.2	NA
**LEAP-012**	Len + Pembro + TACE	46.8	14.6 (0.66)	HR 0.80 (*p* = 0.0867)*
	TACE alone	33.3	10.0	**–**
**TALENTACE**	Atezo + Bev + TACE	49.1	10.3 (0.64)	34.5
	TACE alone	33.9	6.4	35.4

*Data for OS in the LEAP-012 trial were immature at the time of interim analysis

*Abbreviations: Atezo* atezolizumab, *Bev* bevacizumab, *Durva* durvalumab, *HCC* hepatocellular carcinoma, *HR* hazard ratio, Len, lenvatinib; mo, months; mOS, median overall survival; mPFS, median progression-free survival; NA, not analyzed; NR, not reached; ORR, objective response rate; Pembro, pembrolizumab; RECIST, Response Evaluation Criteria in Solid Tumors; TACE, transarterial chemoembolization

While recent trials have demonstrated a significant improvement in PFS, these benefits have not yet translated into a statistically significant OS gain, underscoring the ongoing challenge of optimizing the sequence and synergy of these modalities. Despite the significant PFS gains observed in EMERALD-1 and LEAP-012, interim OS data did not reach statistical significance. This discrepancy likely stems from several critical factors. First, the crossover effect plays a substantial role; the high accessibility and efficacy of subsequent systemic therapies in the control arms after disease progression can significantly dilute the potential OS benefits of the initial combination. Second, the emergence of curative conversion as a viable clinical goal further confounds long-term OS analysis. A significant proportion of patients in the combination arms achieve ‘deep responses’ and transition to radical treatments (e.g., resection or ablation) after successful downstaging, potentially reaching a long-term ‘cancer-free’ state that complicates traditional survival modeling. Finally, these findings raise questions regarding surrogate endpoint validity in the modern era. As post-progression survival extends with potent sequential treatments, mPFS may no longer serve as a robust surrogate for OS, necessitating a re-evaluation of how we define therapeutic success in intermediate-stage HCC trials.

Even though, the effectiveness of immunotherapy combined with TACE has been demonstrated in real-world settings. A multicenter retrospective study aimed to validate the therapeutic efficacy of TACE combined with Atezo/Bev compared to TACE alone in intermediate-stage HCC [47]. After propensity score matching, the combination therapy group showed significantly longer median OS (not reached vs 21.4 months, *p* = 0.008) and PFS (21.7 vs 9.7 months, *p* = 0.009) compared to the TACE-alone group. The combination therapy group also had a higher ORR (66.7% vs 38.1%, *p* = 0.009) and disease control rate (DCR, 92.9% vs 57.1%, *p* < 0.001).

### Curative conversion in immunotherapy-treated intermediate-stage HCC

When the tumor shrinks, many cases can be converted to a cure by resection, ablation, or TACE, resulting in a CR. A multicenter proof-of-concept study conducted in Japan included 110 consecutive patients with Child–Pugh A who received Atezo/Bev as first-line treatment for unresectable and TACE-unsuitable intermediate-stage HCC [48]. Thirty-eight patients (38/110, 35%) achieved clinical or pathological CR, of whom 35 achieved CR with curative conversion. Among the 38 CR patients, 25 (25/110, 23%) achieved drug-free status. However, Kikuchi et al. found that only 8.0% of patients could receive conversion therapy in a real-world setting [49]. Multivariate analysis revealed that BCLC stage was a predictive factor for the implementation of conversion therapy. Nevertheless, intermediate-stage HCC is increasingly viewed as a potentially curable disease, with the exception of cases involving extreme tumor burden—such as bilobar multifocal disease exceeding 10 nodules or those falling beyond the “Up-to-11” criteria. The overarching therapeutic objective for these patients has shifted toward achieving a complete clinical or pathological response (CR), with the ultimate goal of reaching a drug-free status through successful downstaging and subsequent curative intervention [48]. Recently, the Taiwan Liver Cancer Association (TLCA) consensus guidelines for intermediate-stage HCC have endorsed the combination of immunotherapy and TACE as a strategic approach to achieve curative conversion. This recommendation emphasizes utilizing the synergistic effects of locoregional and systemic therapies to downstage patients who were previously ineligible for curative-intent treatments, such as surgical resection or transplantation [13].

## Advanced-stage HCC: optimal treatment selection

**Fig. 1:**
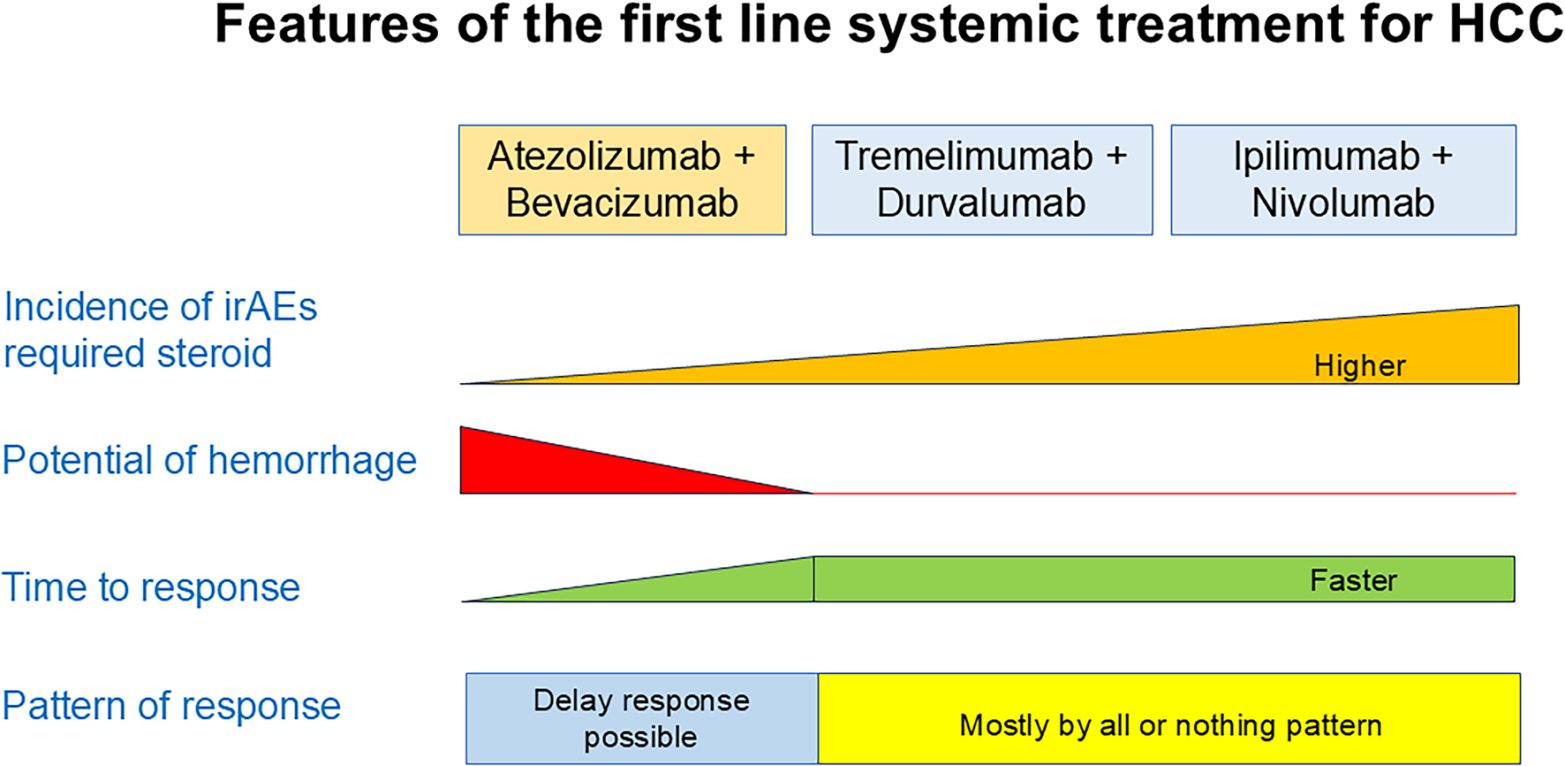
Distinct clinical profiles of first-line systemic combination therapies in hepatocellular carcinoma (HCC). This review figure summarizes the clinical trade-offs between different first-line combination regimens. Atezolizumab plus bevacizumab: characterized by a manageable immune-related adverse event (irAE) profile but a higher risk of hemorrhage due to anti-angiogenic effects. Response kinetics may include delayed patterns. Dual immunotherapy (e.g., STRIDE or Ipi/Nivo): associated with a higher incidence of high-grade irAEs requiring systemic steroids. However, these regimens often achieve a faster time to response, typically exhibiting an “all-or-nothing” efficacy pattern. Abbreviations: *HCC* hepatocellular carcinoma, *irAEs* immune-related adverse events

Currently, the first-line treatment options for advanced HCC are mainly divided into an immunotherapy plus anti-VEGF regimen or a dual immunotherapy regimen. The distinct clinical profiles of these first-line combination therapies—encompassing safety concerns, response kinetics, and efficacy patterns—are schematically summarized in Fig. 1. Specifically, dual immunotherapy regimens are often associated with a higher incidence of immune-related adverse events (irAEs) requiring steroids and faster response kinetics with an “all-or-nothing” pattern. Conversely, the atezolizumab plus bevacizumab combination carries a higher potential for hemorrhage but a relatively manageable irAE profile, with the possibility of delayed response patterns. A landmark study, the IMbrave150 trial, showed that Atezo/Bev resulted in better OS and PFS than SORA for patients with uHCC [50]. After a median 15.6 months of follow-up, the updated data from IMbrave150 showed that the median OS was 19.2 months with Atezo/Bev and 13.4 months with SORA ([HR] = 0.66; *p* < 0.001); and median PFS was 6.9 and 4.3 months in the respective treatment groups ([HR] = 0.65, *p* < 0.001) [51]. Another randomized, open-label, international phase 3 trial compared the efficacy and safety of the anti-PD-1 antibody camrelizumab (Cam) combined with the VEGFR2-targeted TKI rivoceranib (Rivo) vs. SORA as first-line treatment for uHCC [52]. The median OS was significantly prolonged in the Cam/Rivo group compared with SORA (22.1 months vs 15.2 months; [HR] = 0.62; *p* < 0.0001). The median PFS was also significantly increased in the Cam/Rivo group compared with SORA (5.6 months vs 3.7 months; [HR] = 0.52; *p* < 0.0001), providing another new and effective first-line treatment option for this population.

On the other hand, regarding dual immunotherapy regimens, the HIMALAYA study, a global, open-label, phase 3 trial showed that a single, high priming dose of tremelimumab (anti-CTLA4) plus durvalumab (anti-PDL1), an infusion regimen termed STRIDE (single tremelimumab regular interval durvalumab), significantly improved overall survival versus SORA [53]. Regarding the primary objective of OS for STRIDE vs. SORA, the median OS was 16.4 months with STRIDE and 13.8 months with SORA ([HR] = 0.78,* p* = 0.0035). Furthermore, Lau et al. recently reported results from the Asian subgroup of the phase III randomized HIMALAYA study [54]. STRIDE showed better OS compared with SORA ([HR] = 0.68). Especially in Hong Kong and Taiwan (*n* = 141), the OS HR of STRIDE compared with SORA was as high as 0.44. Furthermore, the ORR of STRIDE was numerically higher in the Asian subgroup (28.2%). Finally, another phase 3, open-label, randomized CheckMate 9DW study evaluated the efficacy and safety of Nivolumab (Nivo) + ipilimumab (Ipi) versus LEN or SORA as first-line treatment for patients with uHCC [55]. The median OS was 23.7 months with Nivo + Ipi and 20.6 months with LEN/SORA ([HR] = 0.79; *p* = 0.0180), demonstrating a statistically significant OS benefit with Nivo + Ipi compared with LEN/SORA in patients with untreated uHCC. Table 4 summarizes the efficacy and safety of four first-line immunotherapy-based clinical trials. Notably, the rate of treatment-related adverse events leading to death was highest with the Nivo + Ipi regimen, which may be due to the higher intensity of the dual immunotherapy regimen.

**Table 4:** Efficacy and safety of first-line immunotherapy-based regimens for unresectable HCC

Trial	IMbrave150	CARES-310	HIMALAYA	CheckMate 9DW
Viral etiology, %	70	84	59	63
EHS, %	63	64	53.2	56
VP4, %	14	15 (MVI)	Excluded	Excluded
mOS, months (HR)	19.2 (0.66)	22.1 (0.62)	16.4 (0.78) Asian subgroup: 16.8 (0.68) TW/HK subgroup: 29.4 (0.44)	23.7 (0.79)
mPFS, months (HR)	6.9 (0.65)	5.6 (0.52)	3.8 (0.90)	9.1 (0.87)
ORR, % (CR %)	30.0 (8.0)	25.4 (1.1)	20.1 (3.1) Asian subgroup: 28.2 (2.6) TW/HK subgroup: 33.9 (5.4)	36.0 (7.0)
DCR, %	74.0	78.3	60.1 Asian subgroup: 59.0 TW/HK subgroup: 60.7	68.0
irAE require systemic steroids, %	12.6	16	20.1 (high-dose)	29 (high-dose)
Grades 3–4 hepatic events	Hepatitis: 7%	Immune-mediated hepatitis: 1.5%	Treatment related hepatic SMQ: 5.9%	Immune-mediated hepatitis: 2%
Bleeding Risk, %	Grade 3–4 hemorrhage: 9%	Upper GI hemorrhage: 3%	Grade 3–4 hemorrhage: 0.5%	NA
TRAE-related death, %	2.0	0.4	2.3	4.0

All efficacy outcomes were assessed using RECIST 1.1 criteria

Abbreviations: *CR* complete response, *DCR* disease control rate, *EHS* extrahepatic spread, *HK* Hong Kong, *HR* hazard ratio, *irAE* immune-related adverse event, *mo* months, *mOS* median overall survival, *mPFS* median progression-free survival, *MVI* macrovascular invasion, *NA* not analyzed, *ORR* objective response rate, *RECIST* Response Evaluation Criteria in Solid Tumors, *SMQ* Standardized MedDRA Query, *TRAE* treatment-related adverse event, *TW* Taiwan, *uHCC* unresectable hepatocellular carcinoma, *VP4* main portal vein tumor thrombosis

### Immunotherapy treatment selection

The optimal treatment selection for advanced HCC should be tailored based on the specific clinical presentation, particularly the extent of portal vein tumor thrombus (PVTT) and the presence of extrahepatic metastases (EHS). While PVTT and EHS are both classified as advanced HCC, their management rationales must diverge to address the immediate hemodynamic risks of vascular invasion. Most pivotal trials excluded or limited Vp4 (main portal vein) involvement (Table 4). Vp4 tumor invasion, specifically, represents a distinct clinical entity where the high risk of variceal hemorrhage and hepatic decompensation may necessitate a shift from VEGF-containing regimens toward dual-ICI combinations or the integration of localized radiotherapy (RT/HAIC) to ensure rapid vessel recanalization. The inclusion of Vp1–Vp2 in trials like EMERALD-1 suggests a potential expansion of locoregional–systemic synergies, yet for Vp3–Vp4, the priority remains aggressive systemic control and portal flow preservation.

A structured assessment of safety profiles is essential for balanced therapeutic decision-making, as toxicity spectra differ fundamentally between dual-immune checkpoint inhibitor (ICI) and ICI plus anti-VEGF/TKI combinations (Table 4). While both classes carry comparable risks of high-grade treatment-related adverse events, dual-ICI regimens are characterized by a substantially higher requirement for high-dose systemic corticosteroids to manage immune-mediated toxicities. For instance, high-dose steroids were required in 29% of patients in CheckMate 9DW and 20.1% in the HIMALAYA trial, compared to 12.6% in IMbrave150 and 16% in CARES-310. Furthermore, treatment-related mortality in dual-ICI trials reached 4.0% in CheckMate 9DW and 2.3% in HIMALAYA, compared to 0.4% in CARES-310 and 2.0% in IMbrave150, reflecting the potential severity of intensive immune activation.

In contrast, ICI plus anti-VEGF/TKI regimens carry a distinct risk of hemorrhage, necessitating rigorous pre-treatment endoscopic screening. Grade 3–4 hemorrhage occurred in 9% of patients in IMbrave150 and 3% in CARES-310, whereas the dual-ICI STRIDE regimen demonstrated a markedly lower Grade 3–4 hemorrhage rate of 0.5%. Regarding liver-specific safety, severe hepatic events also vary by regimen, ranging from Grade 3–4 hepatic adverse events (5.9%) in HIMALAYA to immune-mediated hepatitis (2%) and hepatic failure (1%) in CheckMate 9DW. Patients with a marginal functional reserve, such as an ALBI grade 2b or higher, exhibit increased vulnerability to such hepatic decompensation. Consequently, a shift toward more cautious dosing or monotherapy is advised for these patients to preserve hepatic tolerance. By integrating these divergent metrics—steroid-requiring irAE intensity, bleeding risk, and baseline hepatic reserve—clinicians can more effectively tailor immunotherapy to individual patient risk profiles.

**Fig. 2:**
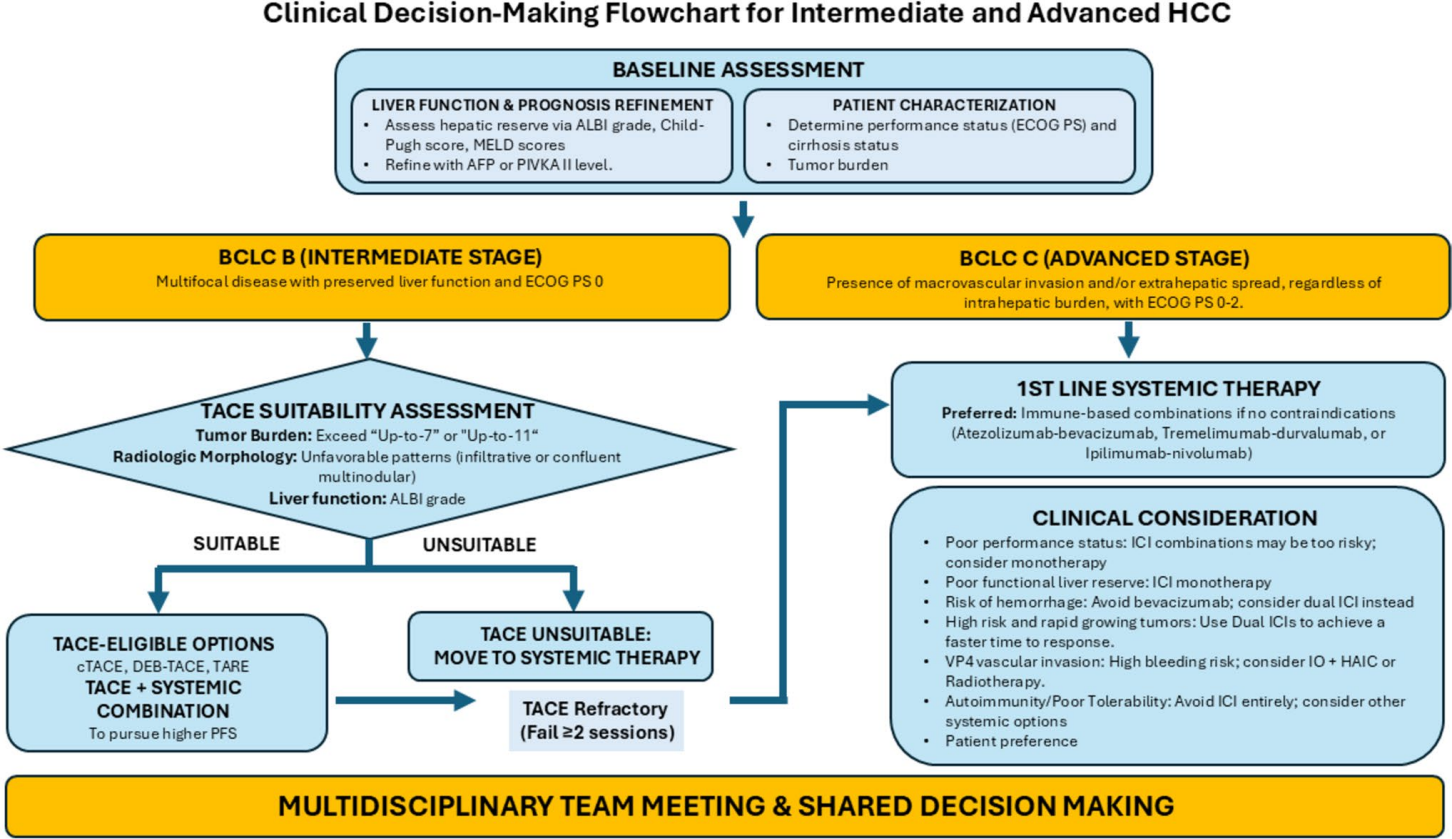
Integrated clinical decision-making algorithm for the management of intermediate-stage and advanced-stage hepatocellular carcinoma (HCC). Comprehensive baseline assessments encompass hepatic reserve, tumor markers, and meticulous patient characterization. For intermediate-stage HCC, treatment selection is rigorously guided by a TACE suitability assessment. Patients presenting with high tumor burden, unfavorable radiologic morphology, or compromised liver function are deemed TACE-unsuitable and should transition directly to systemic therapy. Suitable candidates may receive locoregional options alone or combined with systemic agents to pursue optimal progression-free survival. For advanced-stage disease or TACE-refractory cases, first-line immune-based systemic combinations are preferred if no contraindications exist. Crucially, navigating these complex therapeutic landscapes requires a multidisciplinary team (MDT) approach and shared decision-making (SDM). Abbreviations: *AFP* alpha-fetoprotein, *ALBI* albumin–bilirubin, *BCLC* Barcelona Clinic Liver Cancer, *cTACE* conventional transarterial chemoembolization, *DEB-TACE* drug-eluting bead transarterial chemoembolization, *ECOG PS* Eastern Cooperative Oncology Group performance status, *HCC* hepatocellular carcinoma, *ICI* immune checkpoint inhibitor, *MDT* multidisciplinary team, *MELD* model for end-stage liver disease, *PFS* progression-free survival, *PIVKA II* protein induced by vitamin K absence or antagonist-II, *SDM* shared decision-making, *TACE* transarterial chemoembolization, *TARE* transarterial radioembolization

The selection of an optimal immunotherapy regimen in clinical practice necessitates a nuanced balance of tumor biology and host factors. While dual-ICI regimens, such as the STRIDE or nivolumab/ipilimumab combinations, can induce rapid cytoreduction with a median time to response comparable to VEGF-containing therapies, the latter generally offer a higher disease control rate (DCR). Dual-ICI regimens require careful monitoring for steroid-dependent toxicities. Conversely, ICI-VEGF combinations demand vigilant management of bleeding risks (Fig. 2).

### Curative conversion in advanced-stage HCC

The feasibility of conversion therapy in advanced HCC has been further validated by the TALENTop trial (Sun et al., ESMO 2025). In this study, BCLC stage C patients with macrovascular invasion who achieved a partial response or stable disease after initial atezolizumab plus bevacizumab were randomized to either conversion surgery followed by maintenance therapy or continued systemic treatment. The surgery group achieved a significantly longer median time to treatment failure (TTF) of 20.4 months, compared to 11.8 months in the continuous systemic therapy arm (HR 0.60, *p* = 0.015). While OS data remain immature, a favorable trend was observed in the surgery group (HR 0.67). Furthermore, the grade ≥ 3 surgical complication rate was manageable at 21.7%, with no compromise to surgical feasibility.

The remarkable clinical benefits of conversion surgery in these responders may be underpinned by recent histopathological insights. A multicenter real-world study by Shen et al. demonstrated a significant discrepancy between radiological and pathological responses following atezolizumab plus bevacizumab treatment [56]. Among patients who underwent curative resection for radiologically persistent tumors, a pathological complete response (pCR)—indicating “ghost tumors” with no viable cancer cells—was achieved in 57.7% of PR tumors and 16.7% of SD tumors. Since current imaging modalities are often insufficient to reliably detect PCR cases, surgical intervention not only removes potentially resistant clones but also confirms true disease clearance. These combined findings highlight that for responders to systemic therapy, surgical intervention is a clinically meaningful component of the ‘optimal treatment’ strategy to achieve curative conversion in advanced-stage HCC.

## Biomarker development for HCC immunotherapy

Identifying reliable biomarkers to predict treatment response and survival remains a paramount challenge in HCC management. Although most current evidence for these biomarkers is derived from studies on advanced HCC, their relevance is not limited to the metastatic setting. Molecular signatures and features of the tumor microenvironment (TME)—such as specific genetic mutations, gut microbiota-metabolite axes, and immune-excluded phenotypes—represent fundamental biological determinants of immunotherapy efficacy. Consequently, these biomarkers likely influence therapeutic outcomes across the entire disease spectrum, ranging from neoadjuvant immune priming in early stages to combined locoregional approaches in intermediate stages. Understanding these cross-stage biological drivers is essential for refining patient selection and optimizing the next generation of personalized immunotherapy for all stages of HCC.

### Tumor microenvironment and genetic signature of HCC

The HCC TME often exhibits “immune cold” phenotypes [57]. Luke et al. found that an inverse correlation between β-catenin protein level and T-cell-inflamed gene expression in HCC [57]. The “immune-excluded class,” which accounts for approximately 25–30% of HCC, is characterized by T cell exclusion from the TME and CTNNB1 mutations, and activated Wnt/β-catenin signaling is associated with a lack of T-cell infiltrates [58]. Notably, this disparity was not observed in patients treated with sorafenib (SORA), where PFS remained comparable regardless of Wnt activation status, suggesting that CTNNB1 mutations specifically confer primary resistance to immunotherapy rather than general systemic therapy [59].

Beyond Wnt/β-catenin, the NRF2-COX2-PGE2 axis critically drives the ‘immune-cold’ phenotype. Yamamoto et al. demonstrated that activation of this axis leads to active CD8 + T-cell exclusion, thereby predicting primary resistance to Atezo/Bev. This molecular signature offers a potential therapeutic target, as COX2 inhibition might reverse this immune-excluded environment and restore treatment sensitivity [60].

Molecular correlates of clinical response and resistance to Atezo/Bev in advanced HCC. Zhu et al. reported a comprehensive molecular analysis of tumor samples from 358 patients with HCC participating in the phase 1b GO30140 or phase 3 IMbrave150 trials [61]. Pre-existing immunity was associated with better clinical outcomes with the combination. Reduced clinical benefit was associated with a high ratio of regulatory T cells (Treg) to effector T cells (Teff) and expression of oncofetal genes (GPC3, AFP). The improved efficacy of the combination therapy compared with Atezo alone was associated with higher expression of VEGF receptor 2, Tregs, and myeloid inflammatory signatures [61]. Cappuyns et al. utilized single-cell RNA sequencing (scRNA-seq)-derived signatures to identify distinct molecular subgroups with divergent responses to Atezo/Bev [62]. Their analysis revealed two primary response patterns: the “Immune-competent” and “Angiogenesis-driven” phenotypes, which achieved favorable clinical outcomes. In contrast, the "Resistant" subgroup was characterized by primary resistance to Atezo/Bev. The study identified the accumulation of immunosuppressive myeloid cell types and the activation of the Notch signaling pathway as the key drivers of this therapeutic failure. These findings highlight the importance of the myeloid compartment and Notch activation in mediating immune evasion, providing potential biomarkers for precision patient selection in advanced HCC [62].

A recent study used a multiomic profiling approach to analyze the immune correlatives in patients with HCC treated with STRIDE [63]. Spatial analysis revealed that non-responsive tumors had more Treg cells in adjacent areas rich in immune cells and expressed higher levels of ICOS and PD-1. Specifically, non-responder PD1 + CD8 + T cells within these Treg-enriched niches expressed lower levels of ICOS compared to responders. Furthermore, cell-to-cell communication analysis demonstrated that Treg–CD8 + T interactions were significantly enhanced in non-responder tissues. These findings suggest that a spatially organized, Treg-mediated immunosuppressive network may actively subvert the T-cell activation typically induced by dual CTLA-4 and PD-L1 inhibition [63]. The efficacy of the dual immunotherapy regimen is related to the TME, such as a high CD8 + T cell/Treg ratio in tumor tissue and the amount of activated CD8 + T cells released into the circulation. This dual-compartment response suggests that effective therapy requires both the reversal of local immunosuppression and the successful priming of a systemic immune repertoire [64]. In HCC, anti-CTLA-4 antibodies may deplete Tregs through Fcγ receptor-dependent ADCC activity. Although the mechanism of enhanced binding affinity between the Fcγ region of anti-CTLA-4 and the Fcγ receptors of macrophages/NK cells remains unclear, this could provide predictive biomarkers for dual immunotherapy response [63].

### Non-viral or MASLD-HCC

Non-alcoholic steatohepatitis (NASH) or MASLD is an important driver of HCC. Pfister et al. demonstrated that unconventionally activated, exhausted CD8 + PD-1 + T cells accumulate in livers affected by NASH. In preclinical models of NASH-induced HCC, anti-PD-1 therapy paradoxically expanded this CD8 + PD-1 + population within the tumor without inducing tumor regression, suggesting a state of impaired immune surveillance [65]. These findings have been further validated in clinical trials and real-world cohorts, indicating that non-viral HCC—particularly NASH-HCC—may exhibit diminished responsiveness to immunotherapy. The underlying mechanism appears to be NASH-related aberrant T-cell activation, which causes collateral tissue damage and further compromises the tumor’s immune environment, thereby blunting the efficacy of immune checkpoint inhibition [65]. Ramadori et al. also suggest that in NASH-HCC, autoaggressive CD8 + T cells lost their immune surveillance properties due to their interaction with the metabolic microenvironment [66]. The reduced Th1 responses, increased Treg suppression, and enhanced PD-L1 binding from other cell populations resulted in impaired antitumor activity. However, in a post hoc analysis of IMbrave150 targeting different HCC etiologies, there were no significant differences in ORR, PFS, and OS rates between different etiologies. After Cox regression adjustment, OS and PFS were still not significantly different between NAFLD and other etiologies in multivariable analysis [67]. The effect of immunotherapy on NASH- or MASLD-HCC remains controversial.

### Liver fibrosis and HCC immunotherapy

Scheiner et al. developed and externally validated a scoring system that can predict the prognosis of patients with HCC receiving ICIs [68]. In the training group, baseline serum AFP ≧ 100 ng/ml and CRP ≧ 1 mg/dl were independently associated with worse OS in HCC patients treated with ICIs. The CRAFITY score based on these two variables predicted DCR and OS in the training cohort and was successfully validated in an independent cohort of HCC patients treated with ICIs. Furthermore, a retrospective cohort study investigated the utility of the CRAFITY score in HCC patients treated with Atezo/Bev [69]. The median PFS in the CRAFITY score 0, 1, and 2 groups were 11.8, 6.5, and 3.2 months, respectively (*p* < 0.001); the median OS for patients with CRAFITY scores of 0, 1, and 2 were not reached; 14.3, and 11.6 months, respectively (*p* < 0.001). This score is simple and can be used to predict treatment outcomes and treatment-related adverse events, but prospective validation is needed.

Since most of the HCC patients have underlying liver fibrosis, Mac-2 binding protein glycosylation isomer (M2BPGi) is a biomarker reflecting liver fibrosis status, which may be associated with survival in HCC patients receiving ICIs, particularly in areas where viral hepatitis is prevalent. Lee at el. enrolled 158 patients with unresectable HCC receiving ICI therapy as training cohort and another 60 consecutive patients with unresectable HCC were recruited for validation [70]. In the training group, serum M2BPGi levels were closely correlated with liver fibrosis grade 4, Child–Pugh grade, and ALBI grade. M2BPGi ≥ 1.68 cutoff index (COI) was associated with significantly shorter OS. By integrating baseline alpha-fetoprotein, M2BPGi, portal vein invasion, and non-HBV infection, a new Mac-2 score was developed that effectively stratified patients according to OS (28.8, 13.6, and  8.3 months for scores 0–1, 2, and 3–4, respectively (*p* < 0.001). Compared to the CRAFITY score, ALBI grade, and Child–Pugh classification, the Mac-2 score has demonstrated superior discriminatory power, higher calibration accuracy, and significant net reclassification improvement (NRI). Theoretically, for patients identified with favorable Mac-2-based scores, a more aggressive anti-cancer strategy, including curative-intent resection or intensified systemic therapy should be advocated to maximize survival outcomes.

### Gut microbiota and HCC immunotherapy

Gut microbiota can modulate the outcome of immunotherapy for tumors [71, 72]. Recent studies have shown that the use of antibiotics may interfere with the therapeutic response to ICIs. A retrospective cohort study in Hong Kong included 395 HCC patients who had received ICIs treatment [73]. After propensity score matching, 56 antibiotic users and 99 non-users were further analyzed, and the results showed that the concurrent use of antibiotics and ICIs was associated with higher cancer-related mortality and all-cause mortality [73]. Lee et al. investigated the fecal microbiota and metabolites in patients receiving ICI treatment for uHCC [74]. Before immunotherapy, fecal bacteria were significantly different between OR and progressive disease (PD) patients. Ursodeoxycholic acid (UDCA) and ursocholic acid were significantly enriched in the feces of OR patients and were strongly correlated to the abundance of *Lachnoclostridium*. The coexistence of *Lachnoclostridium* enrichment and *Prevotella* 9 depletion significantly predicted better OS. In the validation cohort, patients with better microbial signatures had better PFS and OS compared with the counter-group. Lee et al. further investigated whether distinct microbiome/metabolomic profiles are associated with the efficacy of combined immunotherapy in patients with MASLD-HCC and viral HCC [75]. MASLD-HCC patients’ fecal flora was dominated by *Bacteroides ovatus, Kluyveromyces georgiensis, Klebsiella oxytoca,* and *Enterococcus faecium*, and had lower levels of short-chain fatty acids (SCFAs) and UDCA than those with viral HCC. Durable responders (DR) in MASLD-HCC were enriched in Bacillus mediterranei ATCC 29149 and had significantly increased levels of SCFAs (acetate, propionate, butyrate, and isobutyrate) and UDCA. In contrast, DR in viral HCC were dominated by *Bifidobacterium* and similarly enriched in SCFAs. In both MASLD-HCC and viral-HCC, fecal acetate levels were a common and significant predictor of DR, PFS, and OS. Patients with higher acetate levels had significantly longer OS and PFS. We concluded that in MASLD-HCC and V-HCC, distinct gut microbiota but shared beneficial metabolites, particularly fecal acetate, were associated with durable efficacy and improved survival with combination immunotherapy. These findings highlight the potential role of gut microbiota and metabolites as biomarkers in predicting the outcome of ICI treatment for HCC, and potentially to modulate the response to immunotherapy.

### AI for HCC immunotherapy

AI and deep learning show remarkable potential in predicting immunotherapy prognosis. A pivotal study of 395 patients treated with various immune checkpoint inhibitors, including nivolumab, pembrolizumab, and ipilimumab demonstrated that machine learning (ML)-based models can predict one-year cancer-specific mortality with high precision. Such predictive capabilities are essential for identifying the patient cohorts most likely to derive a significant therapeutic benefit from immunotherapy [76]. Furthermore, the integration of multi-omics data through AI has enabled the identification of distinct HCC molecular subtypes, each characterized by unique immune profiles and clinical trajectories. For instance, the development of an AI-derived risk score (AIDRS) has allowed for more granular patient stratification, facilitating the prediction of poor outcomes and providing a data-driven framework for personalized immunotherapy decisions [77].

The inherent heterogeneity of HCC and the complexity of its TME have historically hindered the achievement of consistent treatment outcomes. To address this, Gong et al. integrated bulk and single-cell RNA sequencing to construct a “neutrophil-derived signature” (NDS). Developed via machine learning and comprising 10 essential genes, the NDS risk score serves as an independent prognostic factor for OS and a predictive biomarker for the efficacy of both immunotherapy and cytotoxic chemotherapy [78]. Similarly, Li et al. utilized ML integration to develop a T-cell tolerance-derived signature (TCTS) aimed at refining precision therapy. Their findings revealed that patients with high TCTS scores experienced poorer prognoses but exhibited increased sensitivity to oxaliplatin and a superior response to anti-PD-1/PD-L1 immunotherapy. Conversely, the low TCTS group was characterized by fewer genomic alterations, reduced immune activation, and lower PD-L1 expression [79]. Furthermore, a novel cancer stem cell (CSC)-associated HCC cluster was proposed to predict immunotherapy response, utilizing RNA-seq datasets from the TCGA and PCBC. This classification system employs ML algorithms to distinguish between stem cell subtypes, facilitating prognostic stratification and guiding the selection of targeted therapies [80]. Finally, recognizing the role of antigen-presenting cells (APCs) and T-cell infiltration (TCI), Wang et al. leveraged ML to develop long noncoding RNA (lncRNA) signatures related to APCs and TCI. These signatures represent powerful biomarkers for optimizing clinical outcomes and advancing the precision treatment of HCC [81].

ML algorithms are increasingly utilized to analyze multiomics datasets to predict immunotherapy efficacy. Advanced architectures, such as the HCC Multilevel Prognostic Model (HCC-MLPM), integrate clinical, genomic, and immunological data to stratify patients into high- or low-risk cohorts [82]. High-risk patients exhibit a Th2-dominated immune microenvironment, which is associated with a poor response to PD-1/PD-L1 inhibitors. Furthermore, AI can identify immune-related gene signatures (e.g., CXCR2P1, ICOS, TIMD4) and biomarkers such as the Atezo/Bev response signature (ABRS-P), which are associated with prolonged PFS [83]. Deep learning models now predict immune gene activation from histology [84]. Challenges remain, including global validation, protocol standardization, and real-world data integration. Emerging biomarkers and multiomics signatures are summarized in Table 5. To bridge clinical evidence and bedside practice, we propose an integrated algorithm (Fig. 2) for intermediate and advanced HCC. The optimal regimen must be highly individualized, carefully weighing patient-specific factors including performance status, functional liver reserve, tumor burden, risk of hemorrhage, severity of vascular invasion, tolerability to adverse events, underlying autoimmunity, and ultimately, patient preference.

**Table 5:** Emerging biomarkers and multiomics signatures associated with clinical outcomes

Category	Biomarker/model	Mechanism/characteristic	Predictive value/clinical outcome	Comments
Genomic and TME signatures	Wnt/β-catenin pathway	*CTNNB1* mutations; T-cell exclusion (“immune-excluded”)	Primary resistance to ICIs; shorter PFS	Experimental settings
	NRF2-COX2-PGE2 axis	High NRF2 drives CD8 + T-cell exclusion (“immune-cold”)	Resistance to Atezo/Bev; target for COX2 inhibitors	Experimental settings
	Treg/Teff ratio	Balance between immunosuppressive and effector T cells	High ratio predicts reduced benefit from Atezo/Bev	Experimental settings
	Oncofetal genes	High expression of *GPC3* and *AFP*	Associated with poor response to Atezo/Bev	Experimental settings
	Angiogenesis/Notch	High VEGFR2 (favorable) vs. Notch activation (resistance)	Defines molecular subgroups for Atezo/Bev response	Experimental settings
	Spatial Treg network	Enhanced Treg–CD8 + T interactions	Subverts T-cell activation in dual ICI (STRIDE)	Experimental settings
Clinical scores	CRAFITY Score	Integrates serum AFP (≧100 ng/mL) and CRP (≧1 mg/dL)	High score predicts worse OS and DCR in ICIs	Clinically available
	Mac-2 Score	Integrates M2BPGi, AFP, PVI, and non-HBV etiology	Superior discrimination for OS; reflects fibrosis status	Clinically accessible
Gut microbiota	Fecal taxa and SCFAs	*Lachnoclostridium* enrichment; high fecal acetate	Predicts durable response, prolonged PFS, and OS	Clinically accessible
AI and machine learning	NDS Risk Score	Neutrophil-derived signature (10 essential genes)	Independent prognostic factor for OS and ICI efficacy	Requires external validation
	TCTS Score	T-cell tolerance-derived signature (multi-omics)	High score predicts sensitivity to anti-PD-1/PD-L1	Requires external validation
	HCC-MLPM	Multilevel clinical, genomic, and immunological data	Stratifies response based on Th2-dominated TME	Requires external validation

Abbreviations: *AI* artificial intelligence, *AFP* alpha-fetoprotein, *Atezo/Bev* atezolizumab plus bevacizumab, *CRP* C-reactive protein, *DCR* disease control rate, *HBV* hepatitis B virus, *HCC* hepatocellular carcinoma, *HCC-MLPM*, HCC Multilevel Prognostic Model, *ICIs* immune checkpoint inhibitors, *M2BPGi* Mac-2 binding protein glycosylation isomer, *MASLD* metabolic dysfunction-associated steatotic liver disease, *NDS* neutrophil-derived signature, *OS* overall survival, *PD-1/PD-L1* programmed cell death protein 1/ligand 1, *PFS* progression-free survival, *PVI* portal vein invasion, *SCFA* short-chain fatty acids, *TCTS* T-cell tolerance-derived signature, *Teff* effector T cell, *TME* tumor microenvironment, *Treg* regulatory T cell, *VEGFR2* vascular endothelial growth factor receptor 2

In conclusion, the therapeutic landscape for HCC is increasingly characterized by a shift toward personalized medicine. For early-stage HCC, the integration of AI and machine learning models provides a robust framework for predicting postoperative recurrence, enabling more tailored surveillance and adjuvant strategies. In the management of intermediate-stage HCC, a multidisciplinary approach, incorporating locoregional therapies, systemic agents, or intensified combinations has redefined the treatment goal from palliation to curative conversion, aiming for a durable cancer-free state. For advanced HCC, the interplay between the tumor microenvironment, genomic landscapes, gut microbiota, and systemic metabolites has emerged as a critical determinant of response to ICIs. These multifaceted factors serve as pivotal biomarkers that will guide the next generation of precision immunotherapy, ultimately optimizing survival outcomes in this heterogeneous patient population.

## Abbreviations

ABRS-P: Atezolizumab–bevacizumab response signature
ADCC: Antibody-dependent cellular cytotoxicity
AFP: Alpha-fetoprotein
AI: Artificial intelligence
AIDRS: AI-derived risk score
ALBI: Albumin–bilirubin
APCs: Antigen-presenting cells
APPLE: Asia–Pacific Primary Liver Cancer Expert
Atezo: Atezolizumab
AUROC: Areas under the receiver operating characteristic curve
BCLC: Barcelona Clinic Liver Cancer
Bev: Bevacizumab
Cam: Camrelizumab
COI: Cutoff index
CR: Complete response
CRP: C-reactive protein
CSC: Cancer stem cell
DAMPs: Damage-associated molecular patterns
DCR: Disease control rate
DFS: Disease-free survival
DR: Durable responders
Durva: Durvalumab
EFS: Event-free survival
EHS: Extrahepatic metastases
GARSL: Genetic Algorithm for predicting Recurrence after Surgery of Liver cancer
HAIC: Hepatic arterial infusion chemotherapy
HBV: Hepatitis B virus
HCC: Hepatocellular carcinoma
HCC-MLPM: HCC Multilevel Prognostic Model
HCV: Hepatitis C virus
HR: Hazard ratio
ICIs: Immune checkpoint inhibitors
IFN-γ: Interferon-γ
Ipi: Ipilimumab
irAEs: Immune-related adverse events
JSH: Japan Society of Hepatology
LEN: Lenvatinib
lncRNA: Long noncoding RNA
LRT: Locoregional therapy
MASLD: Metabolic dysfunction-associated steatotic liver disease
ML: Machine learning
MPR: Major pathological response
mRECIST: Modified response evaluation criteria in solid tumors
M2BPGi: Mac-2 binding protein glycosylation isomer
MWA: Microwave ablation
NASH: Non-alcoholic steatohepatitis
NDS: Neutrophil-derived signature
Nivo: Nivolumab
NRI: Net reclassification improvement
ORR: Objective response rate
OS: Overall survival
pCR: Pathological complete response
PD: Progressive disease
PD-1/PD-L1: Programmed cell death protein 1/ligand 1
PEI: Percutaneous ethanol injection
Pembro: Pembrolizumab
PFS: Progression-free survival
PR: Partial response
PVTT: Portal vein tumour thrombus
RECICL: Response Evaluation Criteria in Cancer of the Liver
RFA: Radiofrequency ablation
RFS: Recurrence-free survival
Rivo: Rivoceranib
RT: Radiotherapy
SCFAs: Short-chain fatty acids
scRNA-seq: Single-cell RNA sequencing
SD: Stable disease
SORA: Sorafenib
STRIDE: Single tremelimumab regular interval durvalumab
TAAs: Tumor-associated antigens
TACE: Transarterial chemoembolization
TCGA: The Cancer Genome Atlas
TCI: T-cell infiltration
TCTS: T-cell tolerance-derived signature
Teff: Effector T cell
TKI: Tyrosine kinase inhibitor
TLCA: Taiwan Liver Cancer Association
TME: Tumor microenvironment
Treg: Regulatory T cell
TTF: Time to treatment failure
TTUP: Time to untreatable progression
UDCA: Ursodeoxycholic acid
uHCC: Unresectable HCC
VEGFR2: Vascular endothelial growth factor receptor 2
